# Attitude of Medical Students About Their Role and Social Accountability in the COVID-19 Pandemic

**DOI:** 10.3389/fpsyt.2021.645340

**Published:** 2021-06-01

**Authors:** Jihoon Hong, Ikjae Jung, Mingeol Park, Kyumin Kim, Sungook Yeo, Joohee Lee, Sooyeon Suh, Youjin Hong, Jangho Park, Seockhoon Chung

**Affiliations:** ^1^University of Ulsan College of Medicine, Seoul, South Korea; ^2^Department of Psychiatry, Asan Medical Center, University of Ulsan College of Medicine, Seoul, South Korea; ^3^Department of Psychology, Sungshin Women's University, Seoul, South Korea; ^4^Department of Psychiatry, Gangneung Asan Hospital, University of Ulsan College of Medicine, Gangneung, South Korea; ^5^Department of Psychiatry, Ulsan University Hospital, University of Ulsan College of Medicine, Ulsan, South Korea

**Keywords:** COVID-19, pandemic, medical student, medical education, social responsibility

## Abstract

**Background:** In this study, we aimed to explore the attitude of medical students toward their role and social accountability in this pandemic era. An online survey was developed to elicit information on (1) the role of medical students in the pandemic era; (2) Medical education in the “new normal,” and (3) the impact of COVID-19 on medical students.

**Methods:** The online survey, developed by a team consisting of three medical students, three psychiatry residents, and three professors of psychiatry, was conducted on 574 participants (213 medical students, 180 graduates, and 181 professors) in the University of Ulsan College of Medicine, Seoul, South Korea. Anxiety symptom rating scales, including the Stress and Anxiety to Viral Epidemics-6 (SAVE-6) scale and the Generalized Anxiety Disorder−7 (GAD-7) scale, were applied to measure participant anxiety level.

**Results:** Medical students indicated their willingness to join the healthcare response to the COVID-19 pandemic, if requested; however, graduates and professors recommended that medical students continue their training rather than join the pandemic healthcare response. In the new normal era, medical education has had to change appropriately. Moreover, adequate knowledge of COVID-19 infection and spread must be considered for the continuation of clinical clerkships during the pandemic. Overall, medical students who indicated anxiety about treating possible or confirmed cases of COVID-19 rated higher on the SAVE-6 scale. Finally, medical students who reported that COVID-19 had an impact on their studies and daily life rated higher on the general anxiety scale (GAD-7).

**Conclusion:** Social accountability is an important issue for medical students in the pandemic era. At the same time, non-disruption of their academic calendar would ensure continuous availability of component medical professionals, which is important for adequate future healthcare responses.

## Introduction

Coronavirus Disease-19 (COVID-19) is an infectious respiratory disease caused by a novel type of the coronavirus (SARS-CoV-2), which spread throughout China and then around the world since the first case was reported in Wuhan, Hubei Province, in December 2019 (1). In Korea, since the first reported case on January 20, 2020, the COVID-19 crisis level has remained at a “severe” level. Since then, globally, 134,957,021 patients have tested positive for COVID-19 and 2,918,752 patients have died (2). Korea has reported 109,559 cases, with 1,768 deaths due to COVID-19-related causes (3). The COVID-19 pandemic has disrupted the daily lives of people, as the changes that were implemented related not only to the infection itself but also to the general lifestyle. People suffered not only physically but also mentally, and COVID-19 triggered an increase in depression or anxiety among the people (4). The pandemic deeply affected the education field too; with the virus spreading, schools and educational institutions were temporarily closed. Indeed, the educational system itself changed rapidly, as on-site education transformed into non-contact remote teaching and learning through digital media and broadcasts (5).

Past studies show that curriculum of medical students and their daily life undergo multiple changes during times of pandemic, war, or disaster (6–12). These studies indicate that their social role, mental health, and well-being changes during these difficult times. In a survey of Belgian senior medical students at the time of the H5N1 virus outbreak in Europe, 70 per cent responded positively to the idea of taking part in primary health care, and 82.3% of the medical students stated that they would take care of patients who were positive for an epidemic disease (10). Also, in some cases, medical students have taken up roles as frontline healthcare workers (6). For example, during the Spanish Flu of 1918–1919, medical and nursing students supplemented under-staffed medical systems (13). Moreover, during pandemics such as COVID-19, teaching methods must be altered to minimize direct contact between professors and students (12, 14). For example, during the SARS epidemic, the Singapore Medical School developed a web-based learning method for medical students (7). Similarly, many medical schools in Korea are conducting online education during the current coronavirus pandemic. However, problems may arise as there may be some unfamiliarity with online teaching methods among students and professors. Additionally, students may feel uneasy with infectious diseases practices due to delays in learning about infectious diseases (11). Medical students can also be vulnerable to the stressors associated with infectious diseases. The dangers associated with exposure to infectious diseases, such as COVID-19, can often lead to stress among medical staff, as they often fear transmission of the virus to their families and friends (15). This stress can also significantly affect medical students, who are the closest group to a hospital among non-medical personnel. The threats associated with highly infectious diseases are also linked to anxiety symptoms among medical students (11).

Unlike other disasters, infectious diseases directly affect medical students due to their association with hospitals and medical staff. Currently, the role medical students play during a pandemic varies. Classically, The American Association of Medical Colleges (AAMC) defines medical students as “students, not employees,” as students are those who are being given medical knowledge (16). However, in situations like COVID-19, medical students may volunteer to help or support healthcare workers to take COVID-19 samples, since they also have some consciousness as clinicians of the future through practice or volunteer work. In a crisis like a pandemic, the social accountability of medical students, which can be defined as “*the obligation to direct their education, research and service activities toward addressing the priority health concerns of the community*” (17), might become an important issue. This may range from not participating at all to working as frontline health workers. Some medical students have even created Facebook pages to share COVID-19-related information with the public. In this respect, an examination of the societal responsibilities of medical students during a pandemic in this thesis has great significance. It will be also important to assess the stress medical students undergo and the impact of medical education and COVID-19 on their lives. Moreover, so far, there has been little research focusing on the stress of medical students compared with the attention paid to the stress on medical staff and the general public due to infectious diseases. In this regard, it will also be important to assess the stress of medical students and the impact of COVID-19 on their lives. In addition, the survey of online medical education in this study will help direct future medical education decision making during pandemics such as the present one.

Further, student's professionalism is also an important issue to be raised in medical education. Professionalism has been defined as “*constituting those attitudes and behaviors that serve to maintain patient interest above physician self-interest*” (18), and excellence in practice, modesty, recognition of personal limitations, professional judgment, and maintaining a fiduciary relationship with patients (19) are important to medical students in order to not do harm to patients (20). In this study, an online survey was conducted with the cooperation of medical students, graduates, and professors at the University of Ulsan College of Medicine to identify the following three objectives: (1) the role of medical students in the pandemic era, (2) medical education in the “new normal,” and (3) the impact of COVID-19 on medical students. Thus, we explored the attitude and social roles of medical students during the COVID-19 pandemic. Moreover, we investigated the stress experienced by medical students and the impact of the pandemic on their medical education and their lives. The authors believe that this survey will help direct medical education decision making during similar pandemics in the future.

## Methods

### Study Design

This study was conducted using an online survey for medical students during the COVID-19 pandemic between July 23rd and August 1st, 2020. The 213 medical students at the University of Ulsan College of Medicine, Seoul, Korea, participated in the online survey. The survey was conducted by making use of Google Forms® (Google LLC, Mountain View, CA). The questionnaire was distributed to the medical students in their lecture room through a poster, which included the objectives of the study. In Korea, medical students usually take a 2-year pre-medicine curriculum prior to 4 years of medical school. The 3rd and 4th grades of medical students participate in the clinical clerkship. Medical students cannot practice. In this study, 213 of the 247 medical students (86.2%) were in 1st−2nd years of pre-medicine (*N* = 85) and 1st−4th grades (*N* = 162) of the medical school in University of Ulsan College of Medicine. Respondents were rewarded with an e-gift coupon of about 3 dollars. Furthermore, University of Ulsan College of Medicine alumni (180 of 950, 18.9%) and professors (181 of 739, 24.5%) were also requested to respond to the survey as a control group. Written informed consent was waived, because this online survey involved no more than minimal risk to the participants and it may not affect the rights and welfare of the participants adversely, and the study was approved by the Institutional Review Board (2020-1067).

#### Survey Questions

Survey questions were developed by a team consisting of three medical students, three psychiatry residents, and three psychiatry professors. We reviewed the previous literature (6, 9), and developed questions to explore the attitude of medical students about their role during the current pandemic and medical education in the “new normal” situation. The questions focused on three domains of interest, namely, (1) the roles of medical students in the pandemic era, (2) medical education in the “new normal,” and (3) the impact of COVID-19 on medical students.

##### The Role of Medical Students in The Pandemic Era

This section had seven items which elicited information on the attitude medical students have toward their societal duties during the pandemic. The following questions were asked:

A1. How interested are you in COVID-19? (0: never−10: very interested)
A2. How important is social responsibility to medical students? (0: not important−10: very important)
A3. In some countries, medical students work as frontline healthcare workers. Do you feel positive or negative about this? (0: very negative−10: very positive)
A4. Do you think students are ready to be in the medical field in a pandemic situation? (0: never−10: definitely)
A5. If you are requested by a hospital or university to help during the COVID-19 pandemic, are you willing to participate in patient care or assistance? (0: never−10: definitely)
A6. Do you agree with having early graduation or being awarded a temporary license in the event a new infectious disease situation occurs in the future and students are required to enter the medical field quickly? (0: disagree−10: agree)
A7. Do you think you can practice medicine even though you are a trainee? (Yes or No)

##### Medical Education in the “New Normal”

In a pandemic situation, online education often replaces face-to-face education in medical schools. However, since clinical experience in the hospital is also an important part of the medical school curriculum, the change to online education may influence the clinical ability of medical students. Therefore, we asked the following questions:

B1. Do you think that medical school education is working properly during the COVID-19 pandemic? (0: disagree−10: agree)
B2. Do you think you should graduate on schedule and practice medicine, even if your medical school training is not complete due to the ongoing pandemic situation? (0: never−10: definitely)
B3. To what extent do you feel medical school education must change to fit into the new normal era? (0: not needed to be changed−10: definitely to be changed)
B4. How should hands-on clinical practice education be reduced or replaced during the pandemic? (Needs to be reduced vs. maintained)
B5. During COVID-19, how do you think patients will accept students who are trainees? (same as usual/positively/negatively)

##### The Impact of COVID-19 on Medical Students

This part was just for medical students and included questions regarding the influence of COVID-19 on medical student education and daily lives. Therefore, we asked the following questions:

C1. How has your amount of study time changed compared to before the COVID-19 situation? (0: definitely reduced−10: definitely increased)
C2. How has your level of academic-related stress changed compared to before the COVID-19 situation? (0: definitely reduced−10: definitely increased)
C3. How much has the current COVID-19 situation affected your studies? (0: never anxious−10: very anxious)
C4. Are you afraid of interacting with a typical patient (during clinical practice) as a medical student? (0: never anxious−10: very anxious)
C5. Are you afraid of interacting with a suspected coronavirus patient as a medical student (during clinical practice)? (0: never anxious−10: very anxious)
C6. Are you afraid of interacting with a confirmed coronavirus patient as a medical student (during clinical practice)? (0: never affected−10: definitely affected)
C7. How much do you think the current COVID-19 pandemic affects your life and behavior? (0: never affected−10: definitely affected)
C8. How much do you think the current COVID-19 pandemic affects your role as a medical student? (0: never affected−10: definitely affected)

#### Rating Scales

To measure anxiety symptoms of subjects, we asked the following two questions:

1. Do you currently think you are depressed or anxious or do you need help with your mental health?
2. Were you quarantined due to COVID-19 infection?

##### Stress and Anxiety to Viral Epidemics-6 items

The SAVE-6 scale is the anxiety response to a viral epidemic, and a subcategory of the SAVE-9 scale (21), that was initially developed for assessing work-related stress and anxiety responses of healthcare workers to viral epidemics in COVID-19 (22). The SAVE-9 was clustered into the following two factors: (1) anxiety response to viral epidemics and (2) work-related stress. Each item can be rated on a 5-point scale ranging from 0 (never), 1 (rarely), 2 (sometimes), 3 (often), and 4 (always). The cut-off of the total SAVE-9 scale score and anxiety subscale score for higher anxiety levels were found to be 22 and 15, respectively. In this study, only the anxiety response to viral epidemics subcategory (SAVE-6) was applied to all subjects, since it was not appropriate to apply the work-related stress subcategory medical students who are not currently working in the hospital.

##### General Anxiety Disorder−7 Scale

GAD-7 is a seven-item rating scale for assessing general anxiety. Each item can be scored on a four-point Likert scale (0 = not at all to 3 = nearly every day), and the total score of GAD-7 can range from 0 to 21. Cut-off scores for anxiety include 0–4 (minimal), 5–9 (mild anxiety), 10–14 (moderate), and 15–21 (severe) (23).

#### Statistical Analysis

The statistical analyses were done using the SPSS program (Version 21.0 for Windows, IBM Corp., Armonk, NY). The clinical characteristics were presented as mean ± standard deviation. The significance level for all analyses was defined as two-tailed *p* < 0.01. An ANOVA-test with a Scheffe *post-hoc* analysis for continuous variables or a Chi-square test for categorical variables was used for between-group analyses. Spearman correlation was done to explore the relationship between symptom rating scores and the question responses.

## Results

A total of 213 medical students, 180 graduates, and 181 professors at the University of Ulsan College of Medicine participated in this online survey. About 77% (*N* = 445) were male, and 7% (*N* = 41) were now seeking help for depression and/or anxiety. Moreover, about 3% (*N* = 15) were quarantined due to COVID-19 infection. There was no significant difference in general anxiety, as measured by GAD-7, among the three groups. However, a slightly lower level of anxiety was observed in the graduate group regarding viral epidemics, as measured by SAVE-6 (Table 1).

**Table 1:** Demographic characteristics of subjects (*N* = 574).

**Variables**	**Medical students** **(*N* = 213)**	**Graduates** **(*N* = 180)**	**Professors** **(*N* = 181)**	***p*-value**
Sex (male)	150 (70.8%)	155 (86.1%)	140 (77.3%)	0.08
Career year from admission to medical school	3.0 ± 1.7	17.6 ± 8.2	27.9 ± 7.8	<0.01
Now, do you think you are depressed or anxious, or do you need help for your mood state? (Yes) *N* (%)	15 (7.1%)	11 (6.1%)	15 (8.3%)	0.68
Did you experience being quarantined due to infection with COVID-19? (Yes) *N* (%)	2 (0.9%)	8 (4.5%)	5 (2.8%)	0.21
**Symptoms assessments**				
Generalized anxiety disorder-7 (GAD-7)	1.9 ± 3.0	1.6 ± 2.8	1.5 ± 2.7	0.39
Stress and Anxiety to Viral Epidemics-6 items (SAVE-6)	11.0 ± 4.6	10.7 ± 4.3	11.9 ± 4.4	0.02

### The Role of Medical Students in the Pandemic Era

The answers to the questions of the survey questions are shown in Table 2. Additionally, when asked the question “What are the proper roles of medical students during the pandemic?” medical students answers were as followed: “focusing on the medical education and learning as trainees (*N* = 184, 86.3%),” “acting as an informer to neighbors about the COVID-19 (*N* = 132, 62.0%),” “actively learning about the COVID-19 (*N* = 130, 61.0%),” “acting as an assistant for healthcare professionals (*N* = 44, 20.7%),” and “be actively involved in clinical practice, if possible (*N* = 18, 8.5%).” The graduates and professors groups answered “acting as an informer to neighbors about the COVID-19 (*N* = 239, 66.2%),” “focusing on the medical education and learning as trainees (*N* = 220, 60.9%),” “actively learning about the COVID-19 (*N* = 214, 59.3%),” “acting as an assistant for healthcare professionals (*N* = 128, 35.5%),” and “be actively involved in clinical practice, if possible (*N* = 42, 11.6%).”

**Table 2:** Responses of medical students, graduates, and professors to survey questionnaires.

**Survey**	**A. Students** **(*N* = 213)**	**B. Graduates** **(*N* = 180)**	**C. Professors** **(*N* = 181)**	***p-*value**
**Part A. The role of medical students in the pandemic era**				
A1. How interested are you in COVID-19? (0: never~10: very interested)	7.06 ± 1.72	7.63 ± 1.88	8.28 ± 1.69	A < B < C
A2. How important is social responsibility to medical students? (0: not important~10: very important)	7.07 ± 1.81	5.88 ± 2.10	6.95 ± 2.07	A = C > B
A3. In some countries, medical students work as frontline healthcare workers. Do you feel positive or negative about this? (0: very negative~10: very positive)	6.19 ± 2.21	4.99 ± 2.66	5.65 ± 2.57	A > B = C
A4. Do you think students are ready to be in the medical field in a pandemic situation? (0: never~10: definitely)	3.27 ± 2.22	4.17 ± 2.64	4.64 ± 2.22	A < B = C
A5. If you are requested by a hospital or university to help during the COVID-19 pandemic, are you willing to participate in patient care or assistance? (0: never~10: definitely)	7.16 ± 2.04	4.48 ± 3.31	5.80 ± 2.73	A > C > B
A6. Do you agree with having an early graduation or being awarded a temporary license in the event a new infectious disease situation occurs in the future and students are required to enter the medical field quickly? (0: disagree~10: agree)	5.32 ± 2.72	3.31 ± 2.89	3.30 ± 3.07	A > B = C
A7. Do you think you can practice medicine even though you are a trainee? (Yes)	65.2%	48.3%	58.6%	A > C > B
**Part B. Medical education in the “new normal”**				
B1. Do you think that medical school education is working properly during the COVID-19 pandemic? (0: disagree~10: agree)	5.82 ± 2.02	4.81 ± 1.70	4.69 ± 2.03	A > B = C
B2. Do you think you should graduate on schedule and practice medicine, even if your medical school training is not complete due to the ongoing pandemic situation? (0: never~10: definitely)	6.99 ± 1.93	6.96 ± 1.92	6.97 ± 2.08	A = B = C
B3. To what extent do you feel medical school education must change to fit into the “new normal” era? (0: not needed to be changed~10: definitely to be changed)	6.76 ± 1.84	7.02 ± 1.96	7.80 ± 1.49	C > A = B
B4. How should hands-on clinical practice education be reduced or replaced during the pandemic? (Needs to be reduced vs. maintained)	51.9 vs. 48.1%	46.6 vs. 53.4%	52.5 vs. 47.5%	0.47
B5. During COVID-19, how do you think patients will accept students who are trainees? (same as usual/positively/negatively)	52.8/4.7/42%	50.6/6.7/42.8%	50.0/7.2/41.6%	0.27

To the question of “Do you think you can practice medicine even though you are a trainee? (A7),” 65.2% (*N* = 139) of students and 54.6% (*N* = 197) of graduates and professors answered “yes” (*P* < 0.01). The work that medical students can do includes: observation, taking down patient history, patient education, telephone consultation, transfer of test results to patients, case management for discharged patients, and assistance for various procedures.

For those who answered that students should “never be involved in clinical practice,” 79.5% (*N* = 66/83) of medical students explained that the reason they answered that way was due to “lack of ability for clinical practice,” followed by “medical license issue (*N* = 9/83, 10.8%),” and finally “students can be carriers of the virus (*N* = 7/83).” To explain their reasoning why they believe medical students “should never be involved in clinical practice,” graduates and professors answered that it was due to “lack of ability for clinical practice (36.4%, *N* = 64/176),” “medical license issue (*N* = 48/176, 27.3%),” “students can be a carrier for the virus (*N* = 17/176, 9.7%),” and “relevant and appropriate reward for students (*N* = 12/176, 6.8%).”

For the rewards to the medical students for their work, 65.7% (*N* = 377/574) of all subjects answered “grading or credit for volunteering activity” or “monetary compensation (22.1%, *N* = 127/574). Just 9% of medical students and 8.9% of graduates and professors answered “volunteer.”

### Medical Education in the “New Normal”

The answers to the questions in Part B are shown in Table 2.

To the question of “How should hands-on clinical practice education be reduced or replaced during the pandemic? (B4),” 51.9% of medical students, 46.6% of graduates, and 52.5% of professors answered that clinical clerkships should be reduced. As to the reasons for this reduction, 47.4% (*N* = 64/135) of the students answered that they “worry that students will spread the virus” and that it is “not possible for a regular clinical clerkship (24.4%).” However, graduates and professors reasoned that they “worry that students can be infected (38.2%, *N* = 76/199)” and that it is “not possible for a regular clinical clerkship (33.7%).” Additionally, to the question of “How do medical students gain clinical experience in the pandemic era?” 54.9% (*N* = 315) of all respondents answered “*via* meeting the patients,” followed by “*via* meeting standardized patients or medical models (26.3%, *N* = 145),” “online education (12.8%, *N* = 73),” and “virtual reality (4.3%, *N* = 25).”

To the question of “During COVID-19, how do you think patients will accept students who are trainees? (B5),” 52% of students and 49.9% of graduates and professors answered, “same as usual,” but 42% (*N* = 89) of students and 41% (*N* = 148) of graduates and professors answered in the negative.

**Figure 1:**
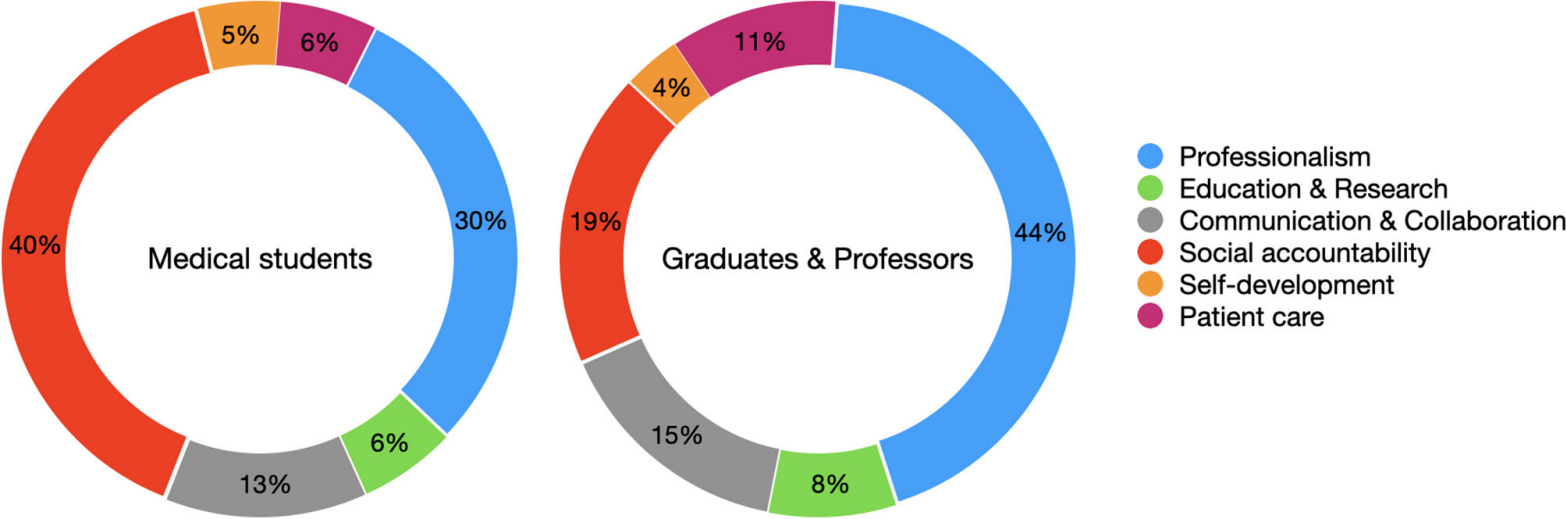
The phase outcome, which is the most important in the pandemic era.

The phase outcomes of this medical school are (1) professionalism, (2) education and research, (3) communication and collaboration, (4) social accountability, (5) self-development, and (6) patient care. Students responded that “social accountability (40%)” is the most important phase outcome, followed by “professionalism (30%),” during the pandemic. However, graduates and professors responded that “professionalism (44%)” is the most important phase outcome, followed by “social accountability (19%),” in the pandemic era (Figure 1).

### Impact of COVID-19 on Medical Students

When we consider the 5-point of each question was neutral, students responded as “no change from previous to after the COVID-19” in the amount of study (C1) and academic stress (C2). When asked about anxiety-related issues while interacting with patients during clinical practice, students responded that they were “afraid” to meet possible (C4, 5.11 ± 2.60) and confirmed (C5, 5.89 ± 2.81) COVID-19 cases. These answers were significantly correlated with the viral epidemic-specific anxiety scale (SAVE-6) score. The impact of COVID-19 on studying (C6), daily life (C7), and social role (C8) of medical students was rated as higher than neutral, and these responses were more significantly correlated with the GAD-7 scores than the SAVE-6 scores (Table 3).

**Table 3:** Correlation coefficients of students' responses to the survey questions with anxiety rating scales scores.

**Survey questions**		**SAVE-6**	**GAD-7**
**Part C. Impact of COVID-19 on medical students**	**Mean ± SD**	**Rho**	**Rho**
C1. How has your amount of study time changed compared to before the COVID-19 situation? (0: definitely reduced~10: definitely increased)	5.00 ± 1.61	–0.03	0.02
C2. How has your level of academic-related stress changed compared to before the COVID-19 situation? (0: definitely reduced~10: definitely increased)	4.93 ± 2.14	0.07	0.09
C3. How much has the current COVID-19 situation affected your studies? (0: never anxious~10: very anxious)	3.93 ± 2.61	0.11	0.03
C4. Are you afraid of interacting with a typical patient (during clinical practice) as a medical student? (0: never anxious~10: very anxious)	5.11 ± 2.60	0.27^**^	–0.04
C5. Are you afraid of interacting with a suspected coronavirus patient as a medical student (during clinical practice)? (0: never anxious~10: very anxious)	5.89 ± 2.81	0.22^**^	−0.03
C6. Are you afraid of interacting with a confirmed coronavirus patient as a medical student (during clinical practice)? (0: never affected~10: definitely affected)	6.30 ± 2.47	0.03	0.16^*^
C7. How much do you think the current COVID-19 pandemic affects your life and behavior? (0: never affected~10: definitely affected)	8.26 ± 1.57	0.03	0.21^*^
C8. How much do you think the current COVID-19 pandemic affects your role as a medical student? (0: never affected~10: definitely affected)	6.24 ± 2.21	0.07	0.21^*^

* *p < 0.05;*

** *p < 0.01*.

## Discussion

In this study, we observed the differences in how medical students and graduates or professors view the role of medical students during the current pandemic. First, the level of interest in COVID-19 was relatively lower in the medical student group compared to the graduate or professor group. Moreover, medical students indicated that they felt that they were not fully qualified to work in a clinical environment during the pandemic. However, they did respond that they would voluntarily aid in healthcare environment when requested. In contrast, graduates and professors believe that medical students are ready to join the frontline of healthcare services, but they still value more education for medical students. Second, in the new normal era, medical students expressed their approval of changes in the medical education system during COVID-19. Specifically, medical students were in favor of a reduction in clinical clerkships during the current pandemic, which was stated to be because of fears associated with transmitting infections to patients. However, while graduates and professors were also in favor of reduced clinical clerkships, they reasoned that the risk of a patient to student transmission, too, was great. Third, medical students who reported being afraid of interacting with possible or confirmed COVID-19 cases also rated as having higher anxiety specific to a viral epidemic (SAVE-6). Medical students who reported that COVID-19-related anxiety influenced their study habits and daily life were also found to have higher general anxiety (GAD-7).

### The Role of Medical Students in the Pandemic Era

Looking at the differences in attitudes toward societal responsibilities between medical students and graduates, we found that, in general, medical students wanted to actively participate in helping society through a medical environment. In contrast, graduates argued that education and professionalism should be fostered rather than social participation. Although, as stated above, medical students showed a lower interest in the coronavirus, they are willing to actively participate in society for three reasons. First, the current medical school curriculum strongly emphasizes learning about societal responsibilities, such as humanities, social sciences, and medical ethics (24). Classical medical education had focused more on medicine itself, while medical education in modern society puts a greater emphasis on not only medicine, but also on morality, humanity skills, and social responsibilities. In this pandemic, medical students can perform an important role based on their medical knowledge, though as students they cannot perform invasive procedures. In accordance with Article 27 (1) 3 of the Medical Service Act in South Korea (25), medical students are required to be guided and supervised by a guidance professor in performance of medical activities related to their major fields. Medical activities include volunteer work to the public or medical activities for a certain period of research. During such medical practice, students shall be marked so that they can recognize that they are students. Likewise, in California, medical-related activities performed by students should be included in approved medical school research courses, where appropriate instructions and supervision by medical school faculty are required. Students who undertake medical practice should state that they are students. In a pandemic, though the activities are limited, they can contribute (26) during review management of and follow-up with COVID-19 patients, and in discussions with patients and patients' families during follow-up calls. They can work in helpline call centers by providing guidance and instructions to people, and aid healthcare professionals (27, 28).

Second, graduates and faculty members tend to worry about participation of students in the medical field due to their own experiences. There is also a possibility that students might have expressed their opinions rather easily because they have not yet had direct experience in the medical field. Graduates and professors, who can encourage the growth of students into independent medical personnel, may have expressed opposition to exposing students to dangerous situations while they are not prepared to engage in activities that take into account students' protection and educational aspects at a medical site. Third, unlike graduates, professors can take a more active stance on promoting societal participation of students, which may underlie the more positive responses of professors regarding the societal participation of medical students (A2, A5, and A7) as compared to the response of graduates. This is likely because, unlike the graduates group, professors not only play the role of health care professionals while treating patients during pandemics, but they also educate and train medical students. As such, the pandemic situation can be an educational opportunity to develop competent and warm-hearted doctors (29).

Social factors that may affect these outcomes should also be considered. It should be considered that opinions may vary depending on how sufficient the workforce, medical resources, medical supplies, etc. of the country's medical personnel are. During COVID-19, Korea has accomplished a relatively successful quarantine due to the extensive medical expertise, powerful workforce, and a sufficient amount of medical supplies. However, lack of medical devices and personal protective equipment in the face of COVID-19 has influenced anxiety and fear among healthcare workers and the sustainability of the health system (30). Moreover, while medical school graduates who are actually actively treating patients may support the idea of medical students participating in the current pandemic response, it may be more effective in the long term for students to focus more on their current studies. This long-term foresight can help Korea be more prepared for future pandemic situations, rather than exposing students to the current dangers. Due to the COVID-19 pandemic, countries that lack sufficient medical staff (the US, South Africa, Brazil) are seeking alternatives, such as early graduation of medical students, use of retired doctors, and permission of state-by-state medical licenses (31, 32). Additionally, easing regulations for medical licenses is also being suggested by medical professionals in those countries (33).

### Medical Education in “New Normal”

Taking into account how various countries have altered their medical school systems in the “new normal” era, graduates in this study indicated, more progressively than medical students or professors, the need for change in the system of medical education. Globally, education is rapidly changing in the wake of the coronavirus crisis, with an increasing need to take into account the various interests and requirements of students. The pandemic induced a rapid transition from conventional medical education to remote on-line education or tele-medicine (34). And it was possible due to the development of digital technologies including immersive technologies (35), though there were issues of digital inequality (36) at the same time. In fact, various educational platforms are being used to combat the loss of access to physical schools during the pandemic. Examples include Coursera, which is led by Stanford (37), and K-MOOC, which is underway in Korea (38). These platforms may be useful for medical school education.

In this study, with regard to a question about the effectiveness of medical education during the COVID-19 pandemic (B1), students favorably viewed the current medical education system than the professors and medical school graduates did. Currently, most medical schools in Korea are offering online lectures or non-contact lectures. In this regard, students who often use online platform-style lectures, dating back to high school, indicate that using online media does not hinder education. However, graduates and professors, who value clinical practice, expressed doubts about the effectiveness of online lectures.

In the case of clinical practice, 54.9% (*N* = 315) of all responses answered: “*via* meeting the patients.” Students, professors, and graduates all agreed that it is critically important for students to interact directly with patients to gain important clinical experience. However, students worried that they would be perceived as transmitters of COVID-19 and, thus, patients would look at them negatively. Consequently, many students responded that clinical practice should be reduced or replaced (51.4%, *N* = 109/211). Students preferred changes in simulated patients and models (24.2%, *N* = 51/211), online learning (11.4%, *N* = 24/211), and virtual reality (4.7%, *N* = 10/211) in future medical practices. Currently, clinical practice during pandemics has checked the results of students' COVID-19 infection tests and has shown passive responses to ensure safety from infectious diseases by himself and the hospital. If COVID-19 is prolonged, or a new global epidemic occurs, medical treatment will need to try various alternatives to physical meetings with patients. Some departments at the Asan Medical Center in Seoul, one of the educational hospitals of the University of Ulsan College of Medicine, introduced online learning in some curricula during student clinical practice. For example, a conference on internal medicine cases for Polyclinic trainees at Asan Medical Center was conducted *via* online lectures. Also, during a pediatric practical course, students attended the course through Skype. Another important issue is how to best utilize face-to-face meetings with patients in a safe environment amid such changes in clinical practice. Finally, an evaluation of medical education according to the changing environment must also be carried out, so that medical students can be educated in a safe environment without degrading the quality of education. It not only seeks a variety of changes to ensure that there is no degradation in quality but also helps to ensure student safety is taken into account (39).

Medical students expressed greater apprehension of directly interacting with COVID-19 suspected patients and COVID-19 confirmed patients, as compared to the general patient population. Since the spread of infectious diseases is regulated by contact, nasal mucus, and airborne transmission, equipment for safe clinical practice must be provided, and clerkship should be conducted. In addition, evaluation and treatment of anxiety and psychological difficulties in the medical student population is critically important during the COVID-19 pandemic. To combat these mental health issues, students should be assigned a counseling office, a professor dedicated to helping with student life, and a homeroom professor.

### Impact of COVID-19 on Medical Students

First of all, our survey indicated that COVID-19 did not have a significant impact on the academic performance or stress levels (C1, C2) of the students. However, COVID-19 did appear to significantly affect academic situations (C6). Specifically, since the method of teaching during the pandemic has changed, the academic burden felt by the medical students was largely unaffected. In this study, the SAVE-6 survey was more correlated with COVID-19-related impacts on clinical practices. Conversely, the GAD-7 survey appeared to represent the impact of COVID-19 on daily life and behavior, as well as academic and social activities. Specifically, the fear of interacting with coronavirus patients during clinical practice, which is generally close to medical practice, was better represented in SAVE-6, a subcategory of SAVE-9 first created for medical staff, than GAD-7. Conversely, GAD-7 better revealed the general lives of students. Through GAD-7, it was revealed that medical students belonged to both the medical staff and the public category for COVID-19. Importantly, this paper used the survey to describe the characteristics of actual medical students and, furthermore, confirmed that COVID-19-related stress has many complex aspects, without a single indicator.

The first limitation of this study was that the survey was developed and distributed to members of a single medical school. We think results may vary depending on a school's academic trends and availability of humanities and social education for medical students.

Second, it should be taken into account that the survey focused on medical students in Korea, which had sufficient medical resources and medical supplies during the COVID-19 pandemic. However, in countries with medical staff shortages, the need for the help of medical students may lead to different results. In addition, it is possible that the Eastern way of thinking, which places a higher value on the protection of students (40) than the Western way of thinking (41), led to graduates and professors expressing their opinions from a more protective perspective.

Lastly, COVID-19-induced anxiety (especially from a living perspective) may have been underreported because the survey was conducted after the social system had stabilized to a certain extent, as opposed to shortly after the COVID-19 outbreak.

## Conclusion

This study attempted to develop a social consensus on the extent to which medical students should play a role within the medical and healthcare system. This included understanding the role of medical students during outbreaks of new infectious diseases, which may aid in them being able to handle pandemics in the future. Moreover, medical school education must be available on new platforms, while the contents of education should also be carried out to recognize public health care and medical care as a part of society, and to cultivate the ability see and be a part of the big picture. In assessing the effects of COVID-19, anxiety about the infection itself and the social, academic, and daily living effects should be assessed on different scales. We believe this paper can be informative for a discussion on the participation of medical students in society by surveying the perception of actual medical students.

## Data Availability Statement

The raw data supporting the conclusions of this article will be made available by the authors, without undue reservation.

## Ethics Statement

The studies involving human participants were reviewed and approved by ASAN Medical Center Institutional Review Board. Written informed consent for participation was not required for this study in accordance with the national legislation and the institutional requirements.

## Author Contributions

YH, JP, SS, and SC: conception or design of the work. JH, IJ, MP, KK, SY, and JL: the acquisition, analysis acquisition, analysis, or interpretation of data. SC: the creation of new software used in the work. JH, IJ, MP, SS, YH, JP, and SC: drafted the work or substantively revised it. All authors have approved the submitted version (and any substantially modified version that involves the author's contribution to the study), agreed both to be personally accountable for the author's own contributions and to ensure that questions related to the accuracy or integrity of any part of the work, even ones in which the author was not personally involved, are appropriately investigated, resolved, and the resolution documented in the literature.

## Conflict of Interest

The authors declare that the research was conducted in the absence of any commercial or financial relationships that could be construed as a potential conflict of interest.

## References

[1] Chan JF Yuan S Kok KH To KK Chu H Yang J. A familial cluster of pneumonia associated with the 2019 novel coronavirus indicating person-to-person transmission: a study of a family cluster. Lancet. (2020) 395:514–23. 10.1016/S0140-6736(20)30154-9 31986261 PMC7159286

[2] World Health Organization. WHO Coronavirus Disease (COVID-19) Dashboard. (2021). Available online at: https://covid19.who.int/ (accessed April 12, 2021).

[3] Coronavirus Disease-19 Republic of Korea. (2021). Available online at: http://ncov.mohw.go.kr/en/ (accessed April 12, 2021).

[4] Xiong J Lipsitz O Nasri F Lui LMW Gill H Phan L. Impact of COVID-19 pandemic on mental health in the general population: a systematic review. J Affect Disord. (2020) 277:55–64. 10.1016/j.jad.2020.08.001 32799105 PMC7413844

[5] Kang B. How the COVID-19 pandemic is reshaping the education service. Future Serv Post COVID-19 Pandemic. (2021) 1:15–36. 10.1007/978-981-33-4126-5_2

[6] Miller DG Pierson L Doernberg S. The role of medical students during the COVID-19 pandemic. Ann Intern Med. (2020) 173:145–46. 10.7326/M20-1281 32259194 PMC7151405

[7] Lim EC Oh VM Koh DR Seet RC. The challenges of “continuing medical education” in a pandemic era. Ann Acad Med. (2009) 38:724–6. 19736579

[8] Slawson RG. Medical training in the United States prior to the civil war. J Evid Based Complement Alternat Med. (2012) 17:11–27. 10.1177/2156587211427404

[9] Valdez-Garcia JE Erana-Rojas IE Diaz Elizondo JA Cordero-Diaz MA Torres-Quintanilla A Esperon-Hernandez RI. The role of the medicine student in COVID-19 pandemic. A shared responsibility. Cir Cir. (2020) 88:399–401. 10.24875/CIRU.M20000066 32567589

[10] Mortelmans LJ De Cauwer HG Van Dyck E Monballyu P Van Giel R Van Turnhout E. Are Belgian senior medical students ready to deliver basic medical care in case of a H5N1 pandemic? Prehosp Disaster Med. (2009) 24:438–42. 10.1017/S1049023X00007287 20066648

[11] Cao W Fang Z Hou G Han M Xu X Dong J. The psychological impact of the COVID-19 epidemic on college students in China. Psychiatry Res. (2020) 287:112934. 10.1016/j.psychres.2020.112934 32229390 PMC7102633

[12] Sharma D Bhaskar S. Addressing the Covid-19 burden on medical education and training: the role of telemedicine and tele-education during and beyond the pandemic. Front Public Health. (2020) 8:589669. 10.3389/fpubh.2020.589669 33330333 PMC7728659

[13] Calm Cool Courageous: Nursing and the 1918 Influenza Pandemic. Available online at: https://www.nursing.upenn.edu/history/publications/calm-cool-courageous/ (accessed April 23, 2021).

[14] Bhaskar S Rastogi A Menon KV Kunheri B Balakrishnan S Howick J. Call for action to address equity and justice divide during COVID-19. Front Psychiatry. (2020) 11:559905. 10.3389/fpsyt.2020.559905 33343410 PMC7744756

[15] Maunder R Hunter J Vincent L Bennett J Peladeau N Leszcz M. The immediate psychological and occupational impact of the 2003 SARS outbreak in a teaching hospital. Canad Med Assoc J. (2003) 168:1245–51. 12743065 PMC154178

[16] Miller DG Pierson L Doernberg S. The role of medical students during the COVID-19 pandemic. Ann Intern Med. (2020) 173:859. 10.7326/L20-1195 33197342

[17] Social Accountability. (2021). Available online at: https://medicaldeans.org.au/priorities/social-accountability/ (accessed April 12, 2021).

[18] Ludmerer KM. Instilling professionalism in medical education. JAMA. (1999) 282:881–82. 10.1001/jama.282.9.881 10478696

[19] Hilton S Southgate L. Professionalism in medical education. Teach Teach Educ. (2007) 23:265–79. 10.1016/j.tate.2006.12.024

[20] Walton M Kerridge I. Do no harm: is it time to rethink the Hippocratic Oath? Med Educ. (2014) 48:17–27. 10.1111/medu.12275 24330113

[21] Stress and Anxiety to Viral Epidemics−6 Items (SAVE-6) for General Population. Available online at: www.save-viralepidemic.net (accessed April 23, 2021).

[22] Chung S Kim HJ Ahn MH Yeo S Lee J Kim K. Development of the stress and anxiety to viral Epidemics-9 (SAVE-9) scale for assessing work-related stress and anxiety in healthcare workers in response to COVID-19. PsyArXiv [Preprint]. (2020). 10.31234/osfio/a52b4 PMC8648611 34873885

[23] Spitzer RL Kroenke K Williams JB Lowe B. A brief measure for assessing generalized anxiety disorder: the GAD-7. Arch Intern Med. (2006) 166:1092–7. 10.1001/archinte.166.10.1092 16717171

[24] Faulkner LR McCurdy RL. Teaching medical students social responsibility: the right thing to do. Acad Med. (2000) 75:346–50. 10.1097/00001888-200004000-00010 10893116

[25] Statutes of the Republic of Korea: Medical Service Act. Available online at: https://elaw.klri.re.kr/eng_service/lawView.do?hseq=40970&lang=ENG (accessed April 12, 2021).

[26] Alshak MN Li HA Wehmeyer GT. Medical students as essential frontline researchers during the COVID-19 pandemic. Acad Med. (2021). 10.1097/ACM.0000000000004056. [Epub ahead of print]. 33735125

[27] Khamees D Brown CA Arribas M Murphey AC Haas MRC House JB. In crisis: medical students in the COVID-19 pandemic. AEM Educ Train. (2020) 4:284–90. 10.1002/aet2.10450 32704600 PMC7369493

[28] Long N Wolpaw DR Boothe D Caldwell C Dillon P Gottshall L. Contributions of health professions students to health system needs during the COVID-19 pandemic: potential strategies and process for U.S. medical schools. Acad Med. (2020) 95:1679–86. 10.1097/ACM.0000000000003611 32701558 PMC7375189

[29] Kalet AL Jotterand F Muntz M Thapa B Campbell B. Hearing the call of duty: what we must do to allow medical students to respond to the COVID-19 pandemic. WMJ. (2020) 119:6–7. 32348064

[30] Bhaskar S Tan J Bogers M Minssen T Badaruddin H Israeli-Korn S. At the epicenter of COVID-19-the tragic failure of the global supply chain for medical supplies. Front Public Health. (2020) 8:562882. 10.3389/fpubh.2020.562882 33335876 PMC7737425

[31] Meet the Medical Students Becoming Doctors in the Middle of a Pandemic. Available online at: https://time.com/5820046/medical-students-covid-19/ (accessed April 23, 2021).

[32] Students Play an Integral Role in Healthcare Delivery: Findings from South Africa. Available online at: https://www.bizcommunity.com/Article/196/858/206054.html (accessed April 23, 2021).

[33] Immigrant Doctors Fight To Contribute To US COVID-19 Response. Available online at: https://www.wpr.org/immigrant-doctors-fight-contribute-us-covid-19-response (accessed April 23, 2021).

[34] Bhaskar S Bradley S Chattu VK Adisesh A Nurtazina A Kyrykbayeva S. Telemedicine across the globe-position paper from the COVID-19 pandemic health system resilience PROGRAM (REPROGRAM) international consortium (part 1). Front Public Health. (2020) 8:556720. 10.3389/fpubh.2020.556720 33178656 PMC7596287

[35] Bhaskar S Bradley S Sakhamuri S Moguilner S Chattu VK Pandya S. Designing futuristic telemedicine using artificial intelligence and robotics in the COVID-19 era. Front Public Health. (2020) 8:556789. 10.3389/fpubh.2020.556789 33224912 PMC7667043

[36] Katz VS Jordan AB Ognyanova K. Digital inequality, faculty communication, and remote learning experiences during the COVID-19 pandemic: a survey of U.S. undergraduates. PLoS ONE. (2021) 16:e0246641. 10.1371/journal.pone.0246641 33566832 PMC7875367

[37] Coursera. Available online at: https://www.coursera.org/ (accessed April 23, 2021).

[38] K-MOOC. Available online at: http://www.kmooc.kr/ (accessed April 23, 2021).

[39] Hjiej G Fourtassi M. Medical students' dilemma during the Covid-19 pandemic; between the will to help and the fear of contamination. Med Educ Online. (2020) 25:1784374. 10.1080/10872981.2020.1784374 32578522 PMC7482721

[40] Chao R Tseng V. Handbook of Parenting: Social Conditions and Applied Parenting. 2nd ed. Mahwah, NJ, US: Lawrence Erlbaum Associates Publishers (2002). p. 59–93.

[41] Stewart S MH B. A critical look at parenting research from the mainstream: problems uncovered while adapting Western research to non-Western cultures. Br J Dev Psychol. (2010) 20:379–92. 10.1348/026151002320620389

